# The Connection between the Averted Infections Ratio and the Rate Ratio in Active-control Trials of Pre-exposure Prophylaxis Agents

**DOI:** 10.1515/scid-2019-0006

**Published:** 2019-07-12

**Authors:** David T. Dunn, David V. Glidden

**Affiliations:** 1 MRC Clinical Trials Unit at UCL, London, UK; 2 Epidemiology & Biostatistics Department, University of California, San Francisco, CA, USA

**Keywords:** hiv, non-inferiority, active-control trials

## Abstract

The design and analysis of active-control trials to evaluate experimental HIV pre-exposure prophylaxis (PrEP) agents pose serious statistical challenges. We recently proposed a new outcome measure, the averted infections ratio (AIR) – the proportion of infections that would be averted by using the experimental agent rather than the control agent (compared to no intervention). The main aim of the current paper is to examine the mathematical connection between AIR and the HIV incidence rate ratio, the standard outcome measure. We also consider the sample size implications of the choice of primary outcome measure and explore the connection between effectiveness and efficacy under a simplified model of adherence.

## 1 Introduction

Late-phase trials of experimental HIV pre-exposure prophylaxis (PrEP) agents are currently designed as active-control trials, with oral TDF-FTC, currently the only drug licensed for this indication, constituting the control regimen. In the absence of a validated surrogate, the primary endpoint is an incident HIV infection (Cutrell et al. 2017; Janes et al. 2019). The standard primary outcome measure, following the approach used in earlier placebo controlled trials, is the HIV incidence rate ratio comparing the experimental and control groups (Cutrell et al. 2017; Donnell et al. 2013). In a recent paper, we pointed out serious difficulties in the interpretation of this measure and described an alternative measure of effectiveness, the averted infections ratio (AIR), based on the concept of averted infections (Dunn et al. 2018). The AIR is interpreted as proportion of infections that would be averted by using the experimental agent rather than the control agent (compared to no intervention). The measure is simple to interpret, has direct clinical and public health relevance, and is a natural preservation-of-effect metric for assessing statistical non-inferiority.

The main aim of this paper is to examine the connection between AIR and the rate ratio in more mathematical detail, and to explain how the AIR allows a reduction in sample size for the same level of statistical power. We also point out a curious feature of the AIR concerning effectiveness and efficacy under a simplified model of adherence.

## 2 Statistical Formulation

Currently, most studies of experimental PrEP agents are designed as non-inferiority trials, where the primary aim is to show that HIV incidence is not unacceptably higher with the experimental agent than with TDF-FTC. This is formally judged by whether the observed confidence limit (lower or upper, as appropriate) for the primary outcome measure exceeds a pre-defined non-inferiority margin. A “preservation of effect” argument is often used as a basis for this margin i. e. to aim to show that the experimental agent preserves a minimum fraction of the effect of TDF-FTC relative to placebo or no treatment (Snapinn and Jiang 2008).

Denote the experimental and control groups by the subscripts E and C, respectively. We also consider a hypothetical placebo group denoted by the subscript P. Let λ (subscripted by E, C, or P) denote the population-average HIV incidence rate (used interchangeably for both the parameter and the estimator), and let Δ denote the non-inferiority margin.

The standard analytical approach considers inference on a log incidence scale, the “natural” parameterisation for the exponential family of distributions (Nelder 1998). Non-inferiority is demonstrated if it is shown (probabilistically) that

(1) $$\text{log}\left(\lambda_{\text{E}}\right)-\text{log}\left(\lambda_{\text{C}}\right)<\left(1-\Delta \right)\left[\text{log}\left(\lambda_{\text{P}}\right)-\text{log}\left(\lambda_{\text{C}}\right)\right]$$

(2) $$\Rightarrow \frac{\text{log}\left(\lambda_{\text{P}}\right)-\text{log}\left(\lambda_{\text{E}}\right)}{\text{log}\left(\lambda_{\text{P}}\right)-\text{log}\left(\lambda_{\text{C}}\right)}>\text{Δ}$$

Denote the expression on the LHS of eq. (2) as STD (shorthand for Standard). Although this formulation is not conventional, it facilitates a comparison with the AIR. The latter is defined by (Dunn et al. 2018):

(3) $$\text{AIR}=\frac{\lambda_{\text{P}}-\lambda_{\text{E}}}{\lambda_{\text{P}}-\lambda_{\text{C}}}$$

The superficial similarity of eqs. (2) and (3) masks a key difference: AIR is essentially a (standardised) rate difference measure and STD essentially a (standardised) rate ratio measure.

Specifying *λ*_P_, the incidence rate that would have been observed in the absence of an intervention, is often not practicable. Another tack is to make inferences via the assumed effectiveness of the control group agent:

(4) $$\theta_{\text{C}}=1-\lambda_{\text{C}}/\lambda_{\text{P}}$$

Re-arranging eq. (4) and substituting in eqs. (2) and (3), STD can be expressed as

(5) $$\text{STD}=1-\frac{\text{log}\left(\lambda_{\text{C}}/\lambda_{\text{E}}\right)}{\text{log}\left(1-\theta_{\text{C}}\right)}$$

and AIR expressed as

(6) $$\text{AIR}=\frac{1-\lambda_{\text{E}}/\lambda_{\text{C}}\left(1-\theta_{\text{C}}\right)}{\theta_{\text{C}}}$$

Eq. (6) reveals an interesting point. Although AIR was formulated as a rate difference based measure, when estimated via *θ*_C_ it becomes a linear function of the rate ratio (experimental group relative to control group) observed in the trial. The remainder of this paper considers inference based on *θ*_C_ rather than *λ*_P_. Also, from this perspective, there is no need to conceptualise constant incidence rates as implied by eqs. (2), (3), and (4). The only underlying assumption is a constant hazard ratio, which can be estimated by Cox regression models with no or little loss in statistical efficiency (Efron 1977).

## 3 Comparison of AIR and STD

We exemplify the difference between AIR and STD using a hypothetical two-arm active-control trial. The following conditions are fixed: (a) equal follow-up in the control and experimental arms (b) 40 HIV endpoints in control arm (c) control agent effectiveness of 60 % (relative to placebo). The number of HIV endpoints in the experimental arm is allowed to vary between 20 and 70 (rate ratio of 0.50 to 1.75).

**Figure 1:**
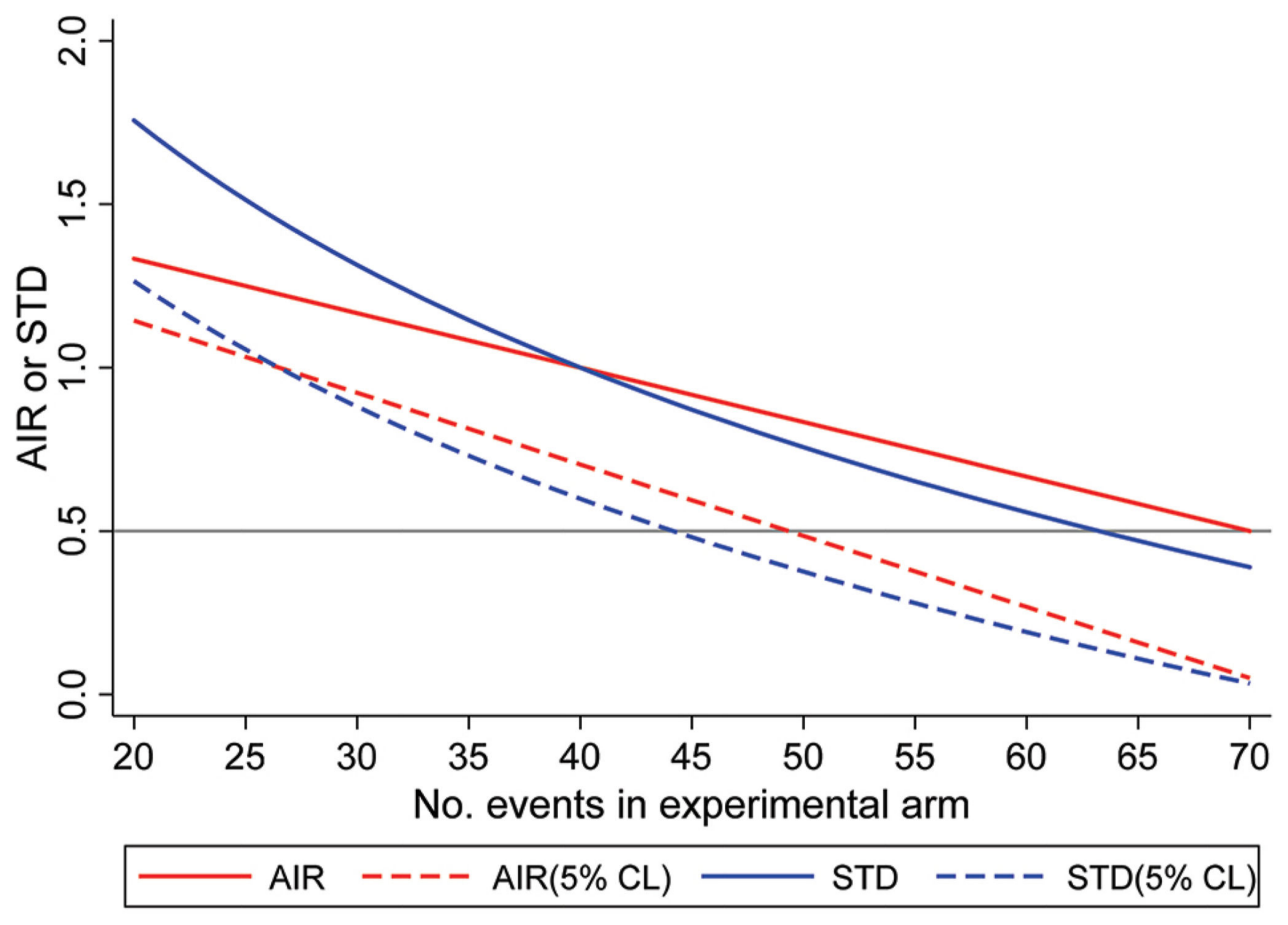
Comparison of point estimates and lower 5 % confidence limit for AIR and STD estimators. Analysis based on a hypothetical two-arm active-control trial (details specified in Section 3).

Figure 1 shows the relationship between AIR and STD and the number of HIV endpoints in experimental arm. Both AIR and STD are equal to one when 40 HIV endpoints are also observed in the experimental arm (i. e. the two agents are equally effective). Both measures are greater than 1 when there are fewer than 40 endpoints in the experimental arm (i. e. it is more effective than the control agent) although the AIR is less than STD. Conversely, the AIR is greater than STD when there are more than 40 endpoints in the experimental arm (i. e. it is less effective than the control agent).

The lower confidence limits, upon which the assessment of non-inferiority is based, are more pertinent than the point estimates. The lower 5 % confidence limits are represented by dotted lines in Figure 1, along with a grey horizontal line representing a non-inferiority margin of 50 %. Focussing on where these lines intercept, non-inferiority is seen to be demonstrated by AIR if there are 49 or fewer HIV endpoints, and by STD if there are 44 or fewer HIV endpoints. That is, if between 45 and 49 HIV endpoints are observed then non-inferiority is demonstrated by AIR but not by STD. This implies that greater statistical power can be achieved by using the AIR rather than the rate ratio. The following section looks at this algebraically.

## 4 Implications for Sample Size by Using the AIR Rather than Rate Ratio

From eq. (5), non-inferiority is demonstrated by the rate ratio if the upper confidence limit for log (*λ*_E_/*λ*_C_) is less than (Δ − 1) log (1 − *θ*_C_). Similarly from eq. (6), non-inferiority is demonstrated by the AIR if the upper confidence limit for log (*λ*_E_/*λ*_C_) is less than log (1 − *θ*_C_Δ) − log (1 − *θ*_C_). It could be questioned whether it is valid to use the same value of Δ for two different metrics but it is not obvious why one should demand a higher or lower preservation of effect with one metric than the other.

As inference regarding non-inferiority is based on log (*λ*_E_/*λ*_C_) in both cases, the two approaches differ only in terms of the non-inferiority margin (on the rate ratio scale). Under the standard statistical approach, the approximate sample size to demonstrate non-inferiority, for a specified power and confidence interval (and assuming a 1:1 allocation ratio), can be shown to be inversely proportional to (Zhu 2016):

(7) $${\left[\left(\Delta -1\right)\text{log}\left(1-\theta_{\text{C}}\right)-\text{log}\left(\lambda_{\text{E}}/\lambda_{\text{C}}\right)\right]}^{2}$$

Similarly, under the AIR approach, the approximate sample size is inversely proportional to

(8) $${\left[\text{log}\left(1-\theta_{\text{C}}\Delta \right)-\text{log}\left(1-\theta_{\text{C}}\right)-\text{log}\left(\lambda_{\text{E}}/\lambda_{\text{C}}\right)\right]}^{2}$$

Thus the ratio of sample sizes (more precisely, the required person-years follow-up) under the standard statistical approach compared with AIR is given by:

(9) $${\left[\frac{\text{log}\left(1-\theta_{\text{C}}\Delta \right)-\text{log}\left(1-\theta_{\text{C}}\right)-\text{log}\left(\lambda_{\text{E}}/\lambda_{\text{C}}\right)}{\left(\Delta -1\right)\text{log}\left(1-\theta_{\text{C}}\right)-\text{log}\left(\lambda_{\text{E}}/\lambda_{\text{C}}\right)}\right]}^{2}$$

In some studies (e. g. HPTN-083 trial comparing injectable cabotegravir versus oral TDF-FTC) the experimental agent is assumed to be more effective than the control agent for the purposes of sample size calculation. However, power is often evaluated (e. g. DISCOVER trial comparing oral TAF-FTC versus oral TDF-FTC) assuming the experimental and control agents are equally effective (*λ*_E_/*λ*_C_ = 1). In this case eq. (9) simplifies to

(10) $${\left[\frac{\text{log}\left(1-\theta_{\text{C}}\Delta \right)-\text{log}\left(1-\theta_{\text{C}}\right)}{\left(\Delta -1\right)\text{log}\left(1-\theta_{\text{C}}\right)}\right]}^{2}$$

**Figure 2:**
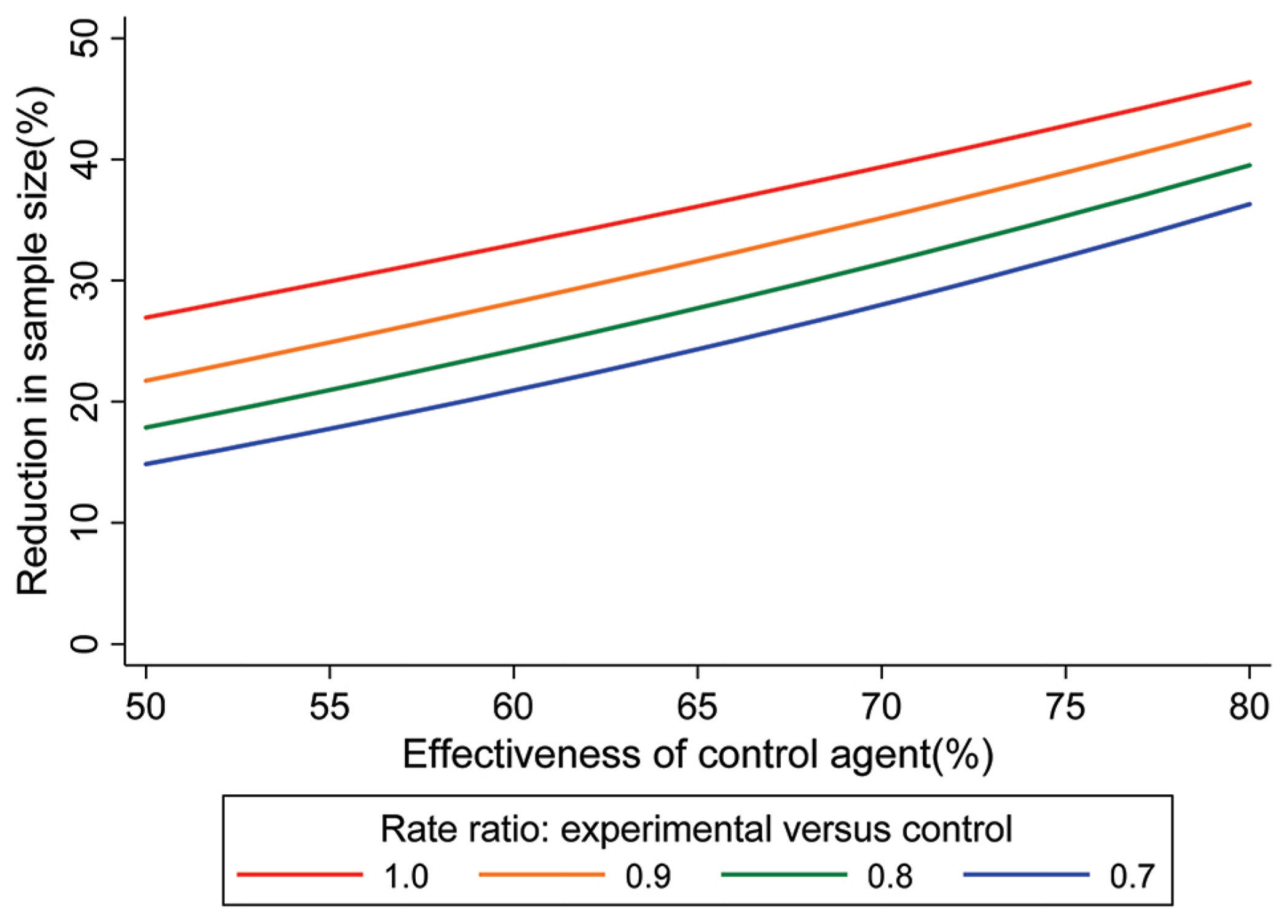
Percentage reduction in sample size achieved by using AIR rather than rate ratio as primary effect measure, according to control drug effectiveness and rate ratio (experimental to control arms). All input parameters assumed equal. Non-inferiority margin (Δ) = 0.5.

Figure 2 shows the percentage reduction in sample size (a simple transformation of the ratio) plotted against *θ*_C_ in the range 0.5–0.8, for Δ = 0.5 (the commonly accepted value for the non-inferiority margin). When *λ*_E_/*λ*_C_ = 1, the reduction ranges between 27 % (*θ*_C_ = 0.5) and 46 % (*θ*_C_ = 0.8). The degree of advantage by using the AIR is diminished when *λ*_E_/*λ*_C_ < 1. For example, when *λ*_E_/*λ*_C_ = 0.7, the reduction ranges between 15 % (*θ*_C_ = 0.5) and 36 % (*θ*_C_ = 0.8). It is emphasised that these are *assumed* values for *λ*_E_/*λ*_C_ which pertain to the sample size calculation. The actual gain in power by using the AIR depends on the *true* value of *λ*_E_/*λ*_C_, which becomes apparent only once the trial is conducted. We note from eq. (9) that the relative sample sizes are independent of *λ*_P_, the underlying HIV incidence rate in the study population, although the absolute sample sizes are dependent on this parameter.

## 5 Efficacy and Effectiveness

There is a key distinction between efficacy and effectiveness (Sommer and Zeger 1991; Dai et al. 2013; Wilder-Smith et al. 2017). Efficacy, the measure of key interest to regulators, is the effect of the intervention under idealised conditions, including taking the drug precisely as prescribed. Effectiveness, of more relevance to public health decision makers, is the effect of the intervention in real-life clinical practice, allowing for imperfect adherence. Our paper thus far has implicitly referred to effectiveness (θ); in this section, we consider the relationship between effectiveness and efficacy for the different effect measures.

For simplicity, we assume that effectiveness is a function of efficacy and adherence only. A meaningful definition of adherence is not straightforward, particularly for “on demand” regimens, since the presence of drug during periods without risky sex is irrelevant (Molina et al. 2015). Again simplifying, in a binary manner, we consider that for each sex act involving exposure to HIV there is: (a) a probability P that there are protective PrEP concentrations, which multiply the risk of acquiring infection by a factor (1- ψ) (b) a probability (1-P) that PrEP concentrations are wholly inadequate and confer *zero* protection against infection. By definition, ψ denotes PrEP efficacy. Our approach has parallels with that of Dai et al. who also invoke a binary division in a counterfactual framework, but splitting participants (rather than sex acts) into compliers and non-compliers (Dai et al. 2013). In a more empirical approach, Hanscom et al. define adherence as the proportion of active-arm participants with detectable levels of PrEP (Hanscom et al. 2018). More realistic, but less tractable, models would allow the level of protection to be a continuous function of PrEP drug concentrations (Anderson et al. 2012).

In our framework, again using the subscripts E, C, and P to denote the experimental, control, and hypothetical placebo groups:

(11) $$\lambda_{\text{C}}=(1-{\text{P}}_{\text{C}})\;\lambda_{\text{P}}+{\text{P}}_{\text{C}}\;(1-\psi_{\text{C}})\;\lambda_{\text{P}}=\lambda_{\text{P}}\;\left(1-{\text{P}}_{\text{C}}\;\psi_{\text{C}}\right)$$

(12) $$\lambda_{\text{E}}=\left(1-{\text{P}}_{\text{E}}\right)\;\lambda_{\text{P}}+{\text{P}}_{\text{E}}\;(1-\psi_{\text{E}})\;\lambda_{\text{P}}=\lambda_{\text{P}}\;\left(1-{\text{P}}_{\text{E}}\;\psi_{\text{E}}\right)$$

In terms of effectiveness,

$$\theta_{\text{C}}=1-\frac{\lambda_{\text{C}}}{\lambda_{\text{P}}}=\frac{1-\lambda_{\text{P}}\;\left(1-{\text{P}}_{\text{C}}\;\psi_{\text{C}}\right)}{\lambda_{\text{P}}}={\text{P}}_{\text{C}}\;\psi_{\text{C}}$$

and similarly for *θ*_E_.

Manipulating eq. (3),

(13) $$\text{AIR}=\frac{{\text{P}}_{\text{E}}\;\psi_{\text{E}}}{{\text{P}}_{\text{C}}\;\psi_{\text{C}}}=\frac{\theta_{\text{E}}}{\theta_{\text{C}}}$$

As we pointed out previously, the AIR can be expressed as the effectiveness of the experimental agent divided by the effectiveness of the active-control agent (Dunn et al. 2018). Surprisingly, it also equal to the ratio of the respective efficacies if P_E_ = P_C_. Thus if adherence is the same for the two agents being compared (which may well be the case for trials evaluating similar oral formulations, such as the DISCOVER trial), the AIR can be interpreted in terms of either effectiveness or efficacy, regardless of the level of adherence in the trial. This does not mean that the level of adherence achieved is irrelevant, however, since this affects the precision of the estimate (higher adherence, more precision).

In contrast both the rate ratio and rate difference depend on the level of adherence, even if this is equal between the two groups.

(14) $$\lambda_{\text{E}}{\text{/λ}}_{\text{C}}=\frac{1-{\text{P}}_{\text{E}}\;\psi_{\text{E}}}{1-{\text{P}}_{\text{C}}\;\psi_{\text{C}}};\lambda_{\text{E}}-\lambda_{\text{C}}=\lambda_{\text{P}}{\;\left({\text{P}}_{\text{C}}\;\psi_{\text{C}}-{\text{P}}_{\text{E}}\;\psi_{\text{E}}\right)}_{}$$

**Figure 3:**
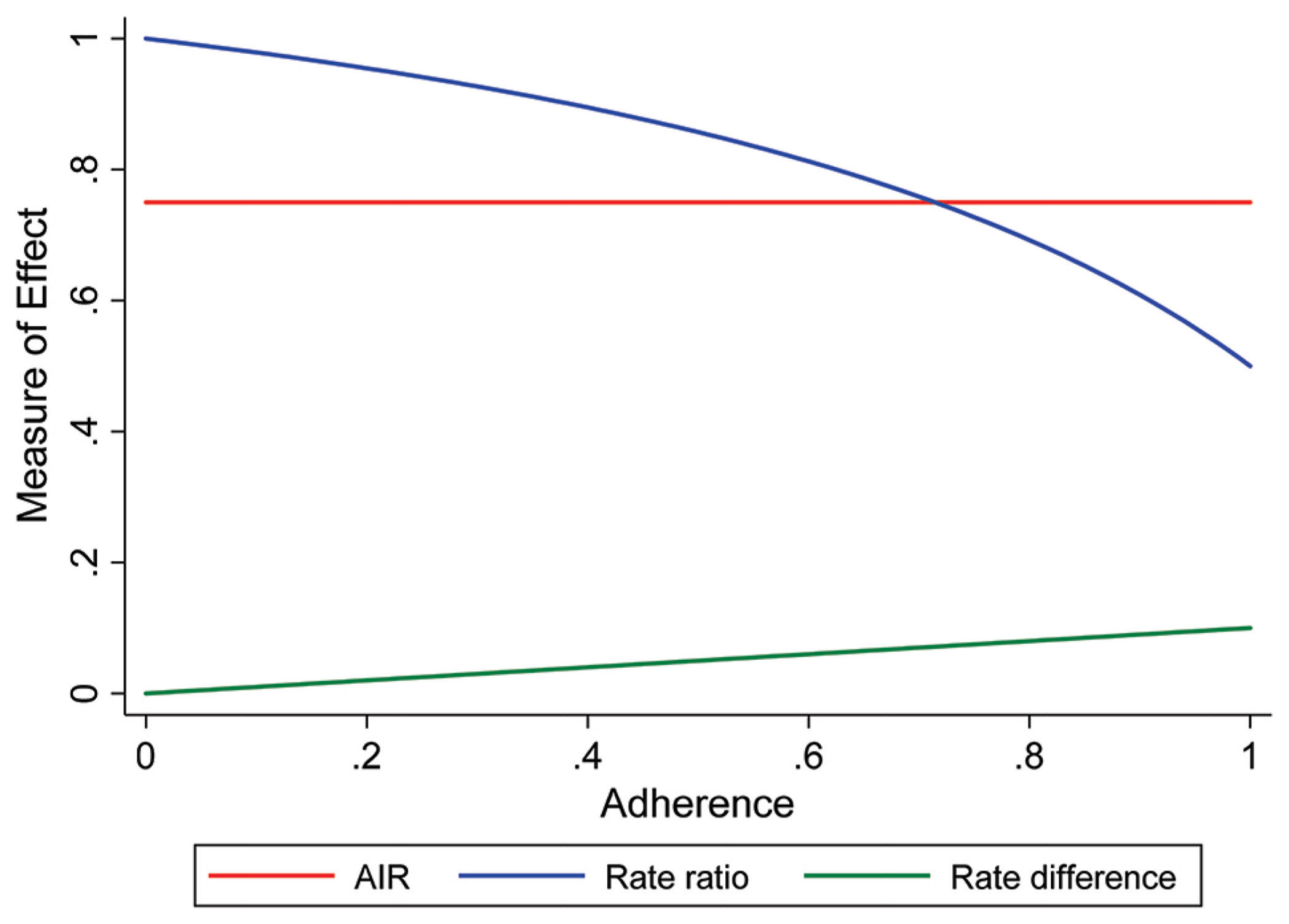
Point estimates of AIR, rate ratio, and rate difference as a function of adherence under a simplified model (see Section 5). Adherence represents the probability of protective PrEP concentrations during each sex act involving exposure to HIV. Footnote. Assumptions: control drug effectiveness = 80 %, experimental drug effectiveness = 60 %, placebo incidence = 5 per 100 PY.

These measures are drawn closer to the null values of one and zero, respectively, the higher the level of non-adherence. Figure 3 shows how the various measures relate to adherence (as quantified by the parameter P described above) when *θ*_C_ = 0.8, *θ*_E_ = 0.6, and *λ*_P_ = 0.05. As predicted by the algebra, the value of AIR is invariant.

## 6 Discussion

In this short note we provide more mathematical detail about the AIR than we presented in our original exposition (Dunn et al. 2018). Apart from ease of interpretation, its adoption allows the use of smaller sample sizes compared with basing inference on the rate ratio. These savings are substantial, typically between 30 % and 40 % for plausible assumptions about the effectiveness of the active control agent. The reduction in sample size may seem like statistical sleight-of-hand, merely being a consequence of using a less stringent non-inferiority margin for the rate ratio. Our counter-argument is that the AIR is a more meaningful scale for assessing preservation of effect. Work in progress on sample size calculations based on the hypothetical placebo incidence (rather than the effectiveness of the control agent) suggests an even greater advantage in using the AIR.

Another interpretational advantage of the AIR is that it reflects preservation of effect for both effectiveness and efficacy for two agents with the same adherence. We stress that this interpretation only applies under our postulated model, which includes simplistic assumptions. Further research is required to test the robustness of this conclusion under different model formulations. Irrespective of the intended analytical approach, there are compelling reasons to attempt to measure adherence within a trial, including generating knowledge to allow development of more realistic causal models and to examine the plausibility of prior assumptions about the effectiveness of the control agent (Dai et al. 2013; Hanscom et al. 2018).

Finally, although the AIR was developed in the context of non-inferiority trials, this does not preclude its application to superiority trials. Indeed, we would argue that it offers the same interpretational advantages over the rate ratio in this setting as well. The gains in statistical power will also hold given that the problems of sample size calculation for non-inferiority and superiority trials are essentially symmetrical (Dunn, Copas, and Brocklehurst 2018).
